# Insulin-like growth factor-binding protein-2 promotes prostate cancer cell growth via IGF-dependent or -independent mechanisms and reduces the efficacy of docetaxel

**DOI:** 10.1038/bjc.2011.127

**Published:** 2011-04-12

**Authors:** C C Uzoh, J M P Holly, K M Biernacka, R A Persad, A Bahl, D Gillatt, C M Perks

**Affiliations:** 1 IGFs and Metabolic Endocrinology Group, School of Clinical Sciences, Learning and Research Building, Southmead Hospital, Bristol, UK; 2 Department of Urology, Bristol Royal Infirmary, University Hospitals Bristol, Bristol, UK; 3 Department of Clinical Oncology, Bristol Haematology and Oncology Centre, University Hospitals Bristol, Bristol, UK; 4 Bristol Urological Institute, Southmead Hospital, Bristol, UK

**Keywords:** IGFBP-2, IGF-independent, *β*-1 integrin, PTEN

## Abstract

**Background:**

The development of androgen independence, chemo-, and radioresistance are critical markers of prostate cancer progression and the predominant reasons for its high mortality. Understanding the resistance to therapy could aid the development of more effective treatments.

**Aim:**

The aim of this study is to investigate the effects of insulin-like growth factor-binding protein-2 (IGFBP-2) on prostate cancer cell proliferation and its effects on the response to docetaxel.

**Methods:**

DU145 and PC3 cells were treated with IGFBP-2, insulin-like growth factor I (IGF-I) alone or in combination with blockade of the IGF-I receptor or integrin receptors. Cells were also treated with IGFBP-2 short interfering ribonucleic acid with or without a PTEN (phosphatase and tensin homologue deleted on chromosome 10) inhibitor or docetaxel. Tritiated thymidine incorporation was used to measure cell proliferation and Trypan blue cell counting for cell death. Levels of IGFBP-2 mRNA were measured using RT–PCR. Abundance and phosphorylation of proteins were assessed using western immunoblotting.

**Results:**

The IGFBP-2 promoted cell growth in both cell lines but with PC3 cells this was in an IGF-dependent manner, whereas with DU145 cells the effect was independent of IGF receptor activation. This IGF-independent effect of IGFBP-2 was mediated by interaction with *β*-1-containing integrins and a consequent increase in PTEN phosphorylation. We also determined that silencing IGFBP-2 in both cell lines increased the sensitivity of the cells to docetaxel.

**Conclusion:**

The IGFBP-2 has a key role in the growth of prostate cancer cells, and silencing IGFBP-2 expression reduced the resistance of these cells to docetaxel. Targeting IGFBP-2 may increase the efficacy of docetaxel.

About 11.9% of cancers in men are due to prostate adenocarcinoma (CaP) and its incidence and prevalence are increasing (Ferlay *et al*, 2010). Androgen deprivation therapy is the mainstay for treating locally advanced, metastatic or recurrent disease. Development of androgen independence (AI), chemo- and radioresistance are critical markers of progression, and the predominant reason for its high cancer-related mortality. When AI occurs, docetaxel-based chemotherapy is one of the few treatment options available. Hence, many are endeavouring to identify molecular factors that could mediate progression to therapy resistance and more lethal metastatic disease. The insulin-like growth factor (IGF) axis, which consists of two ligands (IGF-I and IGF-II) that principally signal via the type 1 IGF receptor (IGF-IR) and a family of six high-affinity IGF-binding proteins (IGFBPs), has an important role in cancer (Pollak, 2008). Research has clearly established that the IGF axis has a very important role in the development and progression of many epithelial cancers, including prostate (Meinbach and Lokeshwar, 2006). At the cellular level the IGF-I receptor appears to have a fundamental role in maintaining the transformed phenotype for many cancer cells (Baserga *et al*, 2003). Recent prospective epidemiology has consistently shown strong associations between circulating IGF-I levels and the subsequent risk of developing prostate cancer (Roddam *et al*, 2008). Individuals with circulating IGF-I levels within the upper end of the normal range are at significantly increased risk of subsequently developing CaP years later. The actions of the IGFs are modulated by a family of high-affinity IGF-binding proteins (IGFBPs 1–6); IGFBPs function to regulate IGF-I- and IGF-II-induced cell signalling in complex ways that involve both inhibiting as well as promoting IGF action. IGF-independent actions of IGFBPs have also been recognised, indicating that IGFBPs can intrinsically modulate aspects of cell growth and survival (Holly and Perks, 2006). The IGFBP-2 is considered a key factor in prostate cancer progression (Degraff *et al*, 2009) with levels of IGFBP-2 being elevated in patients with CaP, both in serum where levels correlate with those of PSA (Kanety *et al*, 1993) and in the tumours (Bubendorf *et al*, 1999). Serum levels are also associated with prognosis (Inman *et al*, 2005). We recently made the novel observation that IGFBP-2 can intrinsically regulate the tumour suppressor gene, *PTEN* (phosphatase and tensin homologue deleted on chromosome 10; Perks *et al*, 2007). PTEN is a phosphoprotein with dual-function protein and lipid phosphatase activity that dephosphorylates the products of phosphatidyl-inositol 3-kinase (PI3K) and focal adhesion kinase and suppresses PI3K/Akt and mitogen-activated protein kinase signalling (Tamura *et al*, 1999; Dahia, 2000; Cully *et al*, 2006), thereby opposing the survival and proliferative actions of many growth factors. PTEN also has an important role in CaP progression, and global expression profiling identified IGFBP-2 as the most significant biomarker for tumour PTEN status (Mehrian-Shai *et al*, 2007). We have investigated the effects of IGFBP-2 on CaP cell proliferation and on the response to docetaxel; as loss of PTEN is a common cause of CaP progression, we examined both DU145 cells in which PTEN is functional and PC3 cells which lack functional PTEN.

## Materials and methods

### Materials

All chemicals and inhibitors were from Sigma-Aldrich (Dorset, UK) unless stated otherwise. Human, recombinant IGF-I and IGFBP-2 peptides were from Gropep (Adelaide, Australia); IGFBP-2 short interfering ribonucleic acid (siRNA) and random sequence-negative control siRNA were from Qiagen Ltd (Crawley, West Sussex, UK). The human *β*_1_-integrin receptor blocking antibody was from Chemicon (Hampshire, UK), and the control mouse IgG antibody was from Dako (Cambridgeshire, UK). A PTEN inhibitor, bpV(HOpic), was from Calbiochem (Nottingham, UK). Tissue culture plastics were from Greiner Labortechnik Ltd (Tyne and Wear, UK). The enhanced chemiluminescence reagents were from Amersham International (Little Chalfont, UK). The BCA protein assay reagent kit was purchased from Pierce (Rockford, IL, USA).

### Cell culture

DU145 (PTEN+) and PC3 (PTEN−) androgen-independent prostate cancer cells were grown and maintained as described previously (Thomas *et al*, 2009).

Trypan blue cell counting and tritiated thymidine incorporation were carried out as described previously (Thomas *et al*, 2009, 2010).

### Transfection with siRNA

CaP cells were placed in 24 well plates (8 × 10^4^ cells per well) in growth media and transfected with siRNA (50 nM final concentration) for IGFBP-2 or with a random sequence negative control siRNA at the same concentration. At 24 h after transfection, cells were then washed with PBS and placed in serum-free media (SFM) for appropriate lengths of time (as described in figure legends) before dosing.

### Western immunoblotting (WIB)

Cells were lysed as described previously (Thomas *et al*, 2010), while conditioned SFM were collected and concentrated using centrifugal filter devices (Millipore, Watford, UK). Lysates were loaded according to protein concentration and equal volumes of supernatants were assessed (to ensure that changes in abundance were not because of the changes in total cell number). The samples were processed as described previously (Thomas *et al*, 2010). Nonspecific binding sites on the membranes were blocked for a minimum of 2 h with either 5% (w/v) milk in Tris-buffered saline/2% Tween (TBST) for probing with anti-GAPDH (1 : 5000) anti-tubulin (1 : 1500; Chemicon) anti-IGFBP-2 (1 : 1000; Santa Cruz Biotechnology, Heidelberg, Germany) anti-PTEN (1 : 750) or 3% BSA for probing with anti-phospho PTEN (1 : 1000; both from New England Biolabs, Herts, UK). After the removal of excess unbound primary antibody with three TBST washes, appropriate secondary antibodies conjugated to peroxidase were added for 1 h. Peroxidase binding was visualised by enhanced chemiluminescence and detected as described previously (Thomas *et al*, 2010).

### RT–PCR

After extracting RNA from cells with Trizol reagent according to manufacturers' instructions (Invitrogen, Paisley, UK), total RNA (0.5 *μ*g) was used for cDNA synthesis using Superscript III First-Strand cDNA synthesis kit (Invitrogen) with random hexamers. RT–PCR was performed to detect mRNA expression of *IGFBP-2* gene. PCR was carried out in a total volume of 10  *μ*l, using 1  *μ*l of cDNA, 0.25 units Taq DNA polymerase, 2.0 mM MgCl_2_, 0.1 mM dNTPs and 0.25 *μ*M of primers. Primer pairs for IGFBP-2 were designed with online software ‘OligoPerfect Designer’ (Invitrogen). The sense primer for IGFBP-2 was (5′-CCTCAAGTCGGGTATGAAGG-3′) and the antisense primer was (5′-ACCTGGTCCAGTTCCTGTTG-3′). The expected amplified fragment for IGFBP-2 was 162 bp. A housekeeping gene, *GAPDH*, was used as an internal control, the sense primer for *GAPDH* gene was (5′-ACAGTCAGCCGCATCTTC-3′), and the antisense primer was (5′-GACAAGCTTCCCGTTCTCAG-3′). The expected amplified fragment for GAPDH was 259 bp. PCR conditions were 94°C for 5 min, followed by 28 cycles at 94°C for 30 s, 60°C for for 1 min and 72°C for 45 s with final extension at 72°C for 10 min. The amplified DNA products were separated on a 2% agarose gel, stained with ethidium bromide, visualised with an ultraviolet transilluminator.

### Data analysis

Data were analysed with SPSS 12.0.1 for Windows using one-way ANOVA applying *post hoc* least significant difference. A statistically significant difference was present at *P*<0.05.

## Results

### Effects of IGFBP-2 on the proliferation of prostate cancer cells

**Figure 1:**
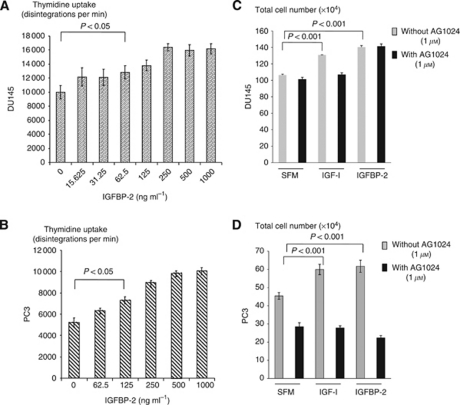
Effects of IGFBP-2 on the proliferation of prostate cancer cells. (**A** and **B**) Graphs show TTI for DU145 (**A**) and PC3 (**B**) cells (0.25 × 10^5^ per well; 24-well plates) treated for 48 h with increasing concentrations of IGFBP-2 (0–1000 ng ml^−1^) after initial plating in GM for 24 h and serum starving for a further 24 h. (**C** and **D**) Graph shows total cell number of DU145 (**C**) and PC3 (**D**) cells (0.2 × 10^6^ per well; six-well plates) pre-dosed for 1 h with 1 *μ*M of AG1024–IGF-IR tyrosine kinase inhibitor or SFM. Cells were then spiked with IGF-I (125 ng ml^−1^) or IGFBP-2 (250 ng ml^−1^) for 48 h after initial plating in GM for 24 h and serum starving for a further 24 h. Graphs are representative of experiments repeated three times showing the mean±s.e.m.

Addition of IGFBP-2 caused a dose-dependent increase in tritiated thymidine uptake in DU145 cells after 48 h, with significant effects occurring from 62.5 ng ml^−1^ IGFBP-2 (*P*<0.05; Figure 1A). This effect was also seen with PC3 cells (Figure 1B). Cell counting produced a similar trend in each of these cell lines (data not shown).

### Is IGFBP-2-induced proliferation dependant on the IGF-I receptor?

We treated both cell lines with a proliferative dose of either IGF-I or IGFBP-2 in the presence or absence of an IGF-IR tyrosine kinase inhibitor, AG1024 (1 *μ*M), which we have shown previously is effective in these cells (Thomas *et al*, 2010). With DU145 cells, IGF-I and IGFBP-2 each induced a significant increase in cell growth (*P*<0.001). AG1024 blocked the response to IGF-I but not that to IGFBP-2 (Figure 1C). With PC3 cells, IGF-I and IGFBP-2 each induced a significant increase in cell growth (*P*<0.001; *P*<0.05, respectively) but in contrast to the DU145 cells, AG1024 blocked the response to both peptides (Figure 1D). This suggested that IGFBP-2 was increasing cell proliferation in an intrinsic, IGF-independent manner in DU145 cells but in an IGF-dependent way in the PC3 cells.

### Involvement of integrin receptors in the intrinsic effects of IGFBP-2 in DU145 cells

**Figure 2:**
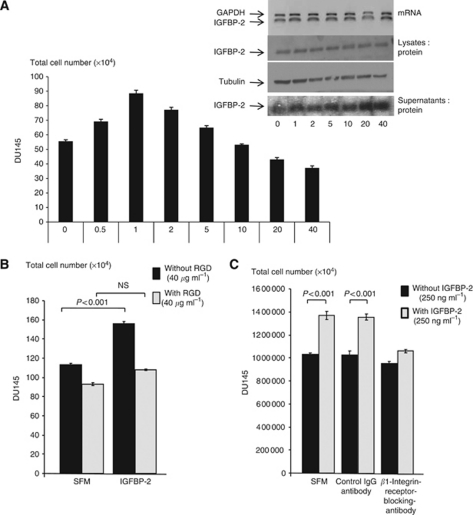
Involvement of integrin receptors in the intrinsic effects of IGFBP-2 on DU145 cells. (**A**) Graph shows total cell number of DU145 cells following treatment with RGD peptide (0–40 *μ*g ml^−1^) for 48 h after initial plating (as in Figures 1A and B) in GM for 24 h and serum starving for a further 24 h. Insert shows a representative PCR blot (of IGFBP-2 m RNA) and WIBs (of IGFBP-2 protein from cell lysates and conditioned cell supernatants). A WIB for tubulin is also shown as a loading control for IGFBP-2 in the cell lysates. (**B**) Graph shows total cell number of DU145 cells pre-dosed for 1 h with RGD peptide (40 *μ*g ml^−1^) or SFM and then re-dosed with SFM or IGFBP-2 (250 ng ml^−1^) for 48 h after initial plating (as in Figures 1C and D) in GM for 24 h and serum starving for a further 24 h. (**C**) Graph shows total cell number of DU145 cells pre-dosed for 1 h with *β*_1_-integrin receptor-blocking antibody (200 ng ml^−1^) or control mouse IgG_1_ antibody (200 ng ml^−1^) or SFM before they were then spiked with IGFBP-2 (250 ng ml^−1^) for 48 h after initial plating (as in Figures 1C and D) in GM for 24 h and serum starving for a further 24 h. All graphs show the mean±s.e.m. of at least three independent experiments each repeated in triplicate.

We tested the effect of a short disintegrin, RGD containing, peptide, which represents an amino acid sequence common to several ECM components that bind to integrins. We observed a dose-dependent decrease in total cell number with increasing RGD peptide concentrations (0–40 *μ*g ml^−1^; Figure 2A). We found that there was no increase in IGFBP-2 mRNA. However, conditioned media from this experiment showed a progressive increase in levels of IGFBP-2 with increasing RGD peptide concentration (Figure 2A insert), suggesting displacement of IGFBP-2 from the cell surface as opposed to an increase in its synthesis. We anticipated that the increase in IGFBP-2 in the cell supernatants would be accompanied by a decrease in the cell lysates. However, levels of IGFBP-2 in the cell lysate remained the same suggesting that the association of the RGD peptide with the cell surface was in some way inhibiting degradation of IGFBP-2. We went on to show that the proliferative effect of exogenous IGFBP-2 was markedly inhibited in the presence of a sub-apoptotic dose of RGD peptide (Figure 2B). This indicated that IGFBP-2 was promoting growth via interaction with an integrin receptor. We then showed that IGFBP-2 acted specifically via integrin receptors containing the *β*_1_ subunit, as a *β*_1_-integrin receptor blocking antibody similarly inhibited cell proliferation stimulated by exogenous IGFBP-2, whereas a control mouse IgG_1_ antibody was without effect (Figure 2C).

### Involvement of PTEN in the intrinsic effects of IGFBP-2 in DU145 cells

**Figure 3:**
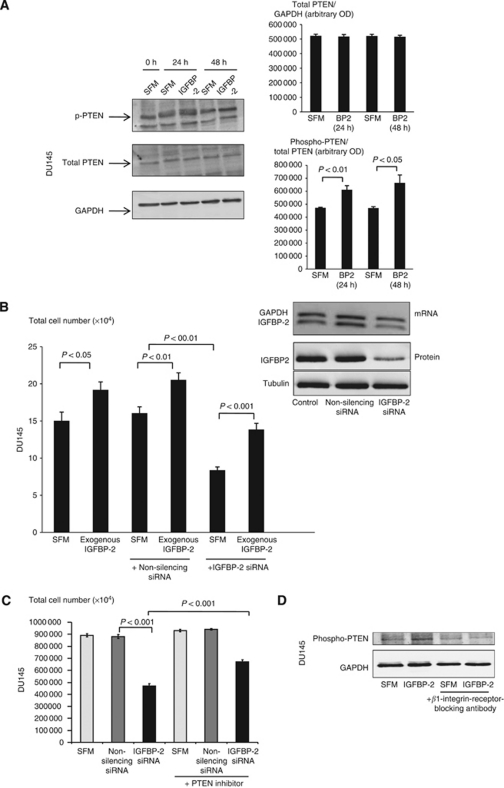
Involvement of PTEN in the intrinsic effects of IGFBP-2 in DU145 cells. (**A**) DU145 cells were seeded (0.25 × 10^6^/T25 flask) in GM for 24 h, serum starved for a further 24 h before re-dosing with SFM or IGFBP-2 (250 ng ml^−1^) for 24 and 48 h and then lysed. A representative WIB (repeated at least three times) for phospho-PTEN, PTEN and GAPDH are shown. The mean±s.e.m. optical densitometry measurements of the three separate experiments are indicated. (**B**) DU145 cells were seeded in GM (0.08 × 10^6^ per well; 24-well plates) and transfected with either IGFBP-2 siRNA (50 nM) or random sequence siRNA (50 nM) for 24 h and then following 4 h in SFM were spiked with or without exogenous IGFBP-2 (250 ng ml^−1^) followed by a further 20 h in SFM before assessment of total cell number. Graph represents the mean±s.e.m. of three individual experiments each repeated in triplicate. Insert (**B**) shows a representative WIB for IGFBP-2 and tubulin protein and RT–PCR for IGFBP-2 mRNA from DU145 cell lysates treated as in **B**. (**C**) DU145 cells were seeded in GM (0.08 × 10^6^ per well; 24-well plates) and transfected as in (**B**) for 24 h and then following 4 h in SFM were spiked with or without a PTEN inhibitor, bpV(HOpic), (0.1 *μ*M) followed by a further 20 h in SFM before cell counting. Graph represents the mean±s.e.m. of three individual experiments each repeated in triplicate. (**D**) DU145 cells were seeded in GM (0.2 × 10^6^ per well; six-well plates) and subjected to SFM conditions for 24 h before pre-dosing for 1 h with a *β*_1_-integrin-receptor-blocking antibody (200 ng ml^−1^) or SFM before they were then spiked with IGFBP-2 (250 ng ml^−1^) for 48 h. Whole-cell lysates were subjected to western immunoblotting for phosphor-PTEN and GAPDH. A representative blot of experiments repeated three times is shown.

We observed that exogenous IGFBP-2 was associated with increased PTEN phosphorylation but without affecting total PTEN abundance. This occurred as early as 10 min (data not shown) following treatment with IGFBP-2 and was sustained for 48 h. With IGFBP-2 being able to induce a 41% (*P*<0.05) increase in phosphorylated PTEN at 48 h (Figure 3A). We established that IGFBP-2 siRNA effectively reduced endogenous levels of IGFBP-2 mRNA and protein, and this correlated with a reduction in tritiated thymidine incorporation, which could be abrogated by adding back exogenous IGFBP-2 (Figure 3B). We also demonstrated that the cell growth inhibitory effect of IGFBP-2 siRNA was reduced in the presence of a specific PTEN inhibitor (0.1 *μ*M), from 47% down to 23% (*P*<0.001), suggesting that this growth inhibition at least partially involved increased PTEN activity (Figure 3C). We have effectively used this PTEN inhibitor previously (Perks *et al*, 2007), and we confirmed that it was effective in this cell line by showing that the IGF-II-induced activation of p-Akt was increased in the presence of the PTEN inhibitor (data not shown). To delineate that PTEN was downstream of IGFBP-2 binding to the *β*_1_-integrin receptor, we examined PTEN phosphorylation following exposure to IGFBP-2 with or without cells being pre-dosed with the *β*_1_-integrin-receptor-blocking antibody. With IGFBP-2 alone, there was an increase in PTEN phosphorylation, the blocking antibody had no effect alone but blocked the IGFBP-2-induced increase in PTEN phosphorylation (Figure 3D). These findings are consistent with IGFBP-2 acting on DU145 cells in an IGF-independent manner via *β*_1_-integrin-receptor-mediated inactivation of PTEN via its phosphorylation.

### Does IGFBP-2 affect sensitivity of CaP to docetaxel-induced cell death?

**Figure 4:**
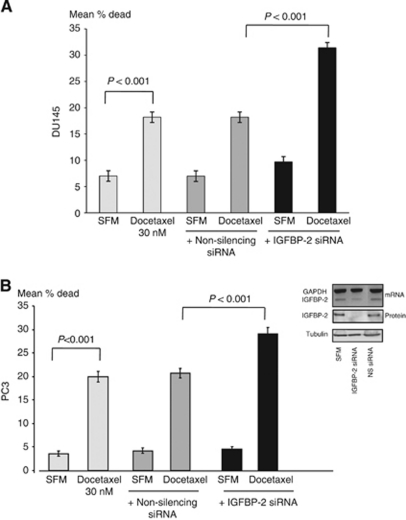
IGFBP-2 affects the sensitivity of CaP to docetaxel. DU145 (**A**) and PC3 (**B**) CaP cells were seeded (0.08 × 10^6^ per well; 24-well plates), respectively, in GM and transfected as in Figure 3B for 24 h. They were then subjected to SFM conditions for a further 24 h and then treated for 24 h with docetaxel (30 nM for each). Cells were then counted and assessed for cell death. The graphs represent the mean±s.e.m. of three experiments each repeated in triplicate. Insert (**B**) shows a representative WIB for IGFBP-2 protein and RT–PCR for IGFBP-2 mRNA from PC3 cell lysates showing effective silencing of IGFBP-2 using siRNA.

We found that docetaxel induced a significant increase in cell death with DU145 cells (*P*<0.001) and with PC3 cells (*P*<0.001) (Figures 4A and B, respectively). The induced cell death was unaffected in the presence of the control, non-silencing siRNA; however, with IGFBP-2 knock down, the response of the cells to docetaxel was increased significantly (*P*<0.001 for both). With DU145 cells docetaxel-induced death increased 1.95-fold and with PC3 cells the induced death increased 1.47-fold. The insert to Figure 4B shows a WIB for IGFBP-2 illustrating effective silencing in PC3 cells. These data showed that removing IGFBP-2 increased the effect of docetaxel to induce CaP cell death.

## Discussion

The development of AI and resistance to therapy remains a challenging issue in the management of prostate cancer. There is therefore a great need for a better understanding of the mechanisms contributing to this progression and identification of new therapeutic targets that may improve effective treatment. One of the established factors that frequently contributes to disease progression is the loss of the tumour suppressor PTEN (Uzoh *et al*, 2009). A screen for markers of PTEN identified IGFBP-2 as being the most significantly associated marker (Mehrian-Shai *et al*, 2007). In addition, there is accumulating evidence that IGFBP-2 may have an important role in prostate cancer progression (Degraff *et al*, 2009). Expression of IGFBP-2 has also been reported to increase following castration and to promote the growth of androgen-independent prostate cancer cells (Kiyama *et al*, 2003).

We have confirmed that IGFBP-2 promoted the growth of DU145 prostate cancer cells and also showed that IGFBP-2 could function as a mitogen for PC3 cells. Our data support previous findings, suggesting that IGFBP-2 may be a key factor in the progression of prostate cancer (Moore *et al*, 2003; Chatterjee *et al*, 2004; Degraff *et al*, 2010). Using an IGF-I receptor inhibitor, we established that although IGF-induced growth was blocked in both cell lines, the proliferative effect of IGFBP-2 was unaffected in the DU145 cells but negated in the PC3 cells. This confirmed that IGFBP-2 could function either in an intrinsic, IGF-independent manner or in an IGF-dependent manner to promote prostate cancer cell proliferation. Enhancement of IGF-mediated effects by IGFBPs can occur via a number of mechanisms, including prolonging IGF half-life, and hence cell exposure or by sequestering IGFs at the cell surface and increasing levels in the vicinity of cell receptors (Perks and Holly, 2008). To date, the mechanism by which IGFBP-2 exerts intrinsic proliferative effects in DU145 cells has not been identified (Chatterjee *et al*, 2004). It had previously been reported that an antibody to IGFBP-2 inhibited the growth of DU145 cells, indicating that IGFBP-2 produced within the cells, had to be secreted in order to promote growth via an autocrine, and not via an intracrine, mechanism (Chatterjee *et al*, 2004). IGFBP-2 contains an integrin recognition sequence, RGD, near the COOH C-terminus, which is the minimum requirement for interaction with integrin receptors. It is well established that integrin receptors contribute to survival and proliferative signals and to the metastatic potential of tumour cells (Schwartz, 1997; Kumar, 1998). One of the most abundant integrins found on DU145 prostate cancer cells is the *β*1, whose expression was reported to be increased with increasing Gleason grade of prostate cancer (Murant *et al*, 1997; Skogseth *et al*, 2006). It has been shown that IGFBP-2 is able to exert intrinsic effects on a Ewing sarcoma, and on a breast cancer, cell line by binding to cell surfaces through its RGD recognition sequence (Schutt *et al*, 2004). We have now shown that the mitogenic actions of IGFBP-2 on DU145 cells were blocked by a short RGD-containing disintegrin peptide or by a *β*_1_-integrin receptor blocking antibody; suggesting that the intrinsic actions of IGFBP-2 are mediated by binding to *β*-1-containing integrin receptors. This was associated with increased phosphorylation of PTEN without affecting total PTEN abundance; the phosphorylation of PTEN has been reported to reduce its phosphatase activity (Uzoh *et al*, 2009). We recently identified IGFBP-2 as a novel regulator of PTEN in breast cancer cells via its interaction with integrin receptors although in those cells the abundance of PTEN protein was reduced (Perks *et al*, 2007). PTEN has been shown to inhibit integrin-mediated signalling (Gu *et al*, 1998), indicating a potential feedback regulation loop. The growth inhibitory effect of IGFBP-2 siRNA was reduced in the presence of a PTEN inhibitor, suggesting that this growth inhibition involved increased PTEN activity. We also found that loss of IGFBP-2 improved the effectiveness of docetaxel-induced cell death. Chemotherapy with docetaxel is the most effective treatment available for metastatic castration-resistant prostate cancer. Our data show that loss of IGFBP-2 reduced the resistance of CaP cells to docetaxel; therefore, targeting IGFBP-2 as an adjunct to this treatment may increase the efficacy of this drug.

Progression of prostate cancer to the development of AI, chemo- and radioresistance remain challenging clinical problems. The search for markers of this progression have revealed IGFBP-2 and PTEN to be strongly associated; our findings provide a mechanistic link between these two putative markers which identifies potential new targets for improving therapeutic response. In PC3 cells, which lack a functional PTEN, IGFBP-2 interestingly retained its ability to promote growth and confer chemoresistance, but in these cells IGFBP-2 acted in a more conventional manner via its ability to enhance IGF-receptor activation.

## References

[bib1] Baserga R, Peruzzi F, Reiss K (2003) The IGF-1 receptor in cancer biology. Int J Cancer 107: 873–877 14601044 10.1002/ijc.11487

[bib2] Bubendorf L, Kolmer M, Kononen J, Koivisto P, Mousses S, Chen Y, Mahlamaki E, Schraml P, Moch H, Willi N, Elkahloun AG, Pretlow TG, Gasser TC, Mihatsch MJ, Sauter G, Kallioniemi OP (1999) Hormone therapy failure in human prostate cancer: analysis by complementary DNA and tissue microarrays. J Natl Cancer Inst 91: 1758–1764 10528027 10.1093/jnci/91.20.1758

[bib3] Chatterjee S, Park ES, Soloff MS (2004) Proliferation of DU145 prostate cancer cells is inhibited by suppressing insulin-like growth factor binding protein-2. Int J Urol 11: 876–884 15479293 10.1111/j.1442-2042.2004.00898.x

[bib4] Cully M, You H, Levine AJ, Mak TW (2006) Beyond PTEN mutations: the PI3K pathway as an integrator of multiple inputs during tumorigenesis. Nat Rev Cancer 6: 184–192 16453012 10.1038/nrc1819

[bib5] Dahia PL (2000) PTEN, a unique tumor suppressor gene. Endocr Relat Cancer 7: 115–129 10903528 10.1677/erc.0.0070115

[bib6] Degraff DJ, Aguiar AA, Chen Q, Adams LK, Williams BJ, Sikes RA (2010) Androgen mediated translational and postranslational regulation of IGFBP-2 in androgen-sensitive LNCaP human prostate cancer cells. Am J Transl Res 2: 200–208 20407609 PMC2855632

[bib7] Degraff DJ, Aguiar AA, Sikes RA (2009) Disease evidence for IGFBP-2 as a key player in prostate cancer progression and development of osteosclerotic lesions. Am J Transl Res 1: 115–130 19956425 PMC2776314

[bib8] Ferlay J, Parkin DM, Steliarova-Foucher E (2010) Estimates of cancer incidence and mortality in Europe in 2008. Eur J Cancer 46: 765–781 20116997 10.1016/j.ejca.2009.12.014

[bib9] Gu J, Tamura M, Yamada KM (1998) Tumor suppressor PTEN inhibits integrin- and growth factor-mediated mitogen-activated protein (MAP) kinase signaling pathways. J Cell Biol 143: 1375–1383 9832564 10.1083/jcb.143.5.1375 PMC2133067

[bib10] Holly J, Perks C (2006) The role of insulin-like growth factor binding proteins. Neuroendocrinology 83: 154–160 17047378 10.1159/000095523

[bib11] Inman BA, Harel F, Audet JF, Meyer F, Douville P, Fradet Y, Lacombe L (2005) Insulin-like growth factor binding protein 2: an androgen-dependent predictor of prostate cancer survival. Eur Urol 47: 695–702 15826765 10.1016/j.eururo.2004.12.015

[bib12] Kanety H, Madjar Y, Dagan Y, Levi J, Papa MZ, Pariente C, Goldwasser B, Karasik A (1993) Serum insulin-like growth factor-binding protein-2 (IGFBP-2) is increased and IGFBP-3 is decreased in patients with prostate cancer: correlation with serum prostate-specific antigen. J Clin Endocrinol Metab 77: 229–233 7686915 10.1210/jcem.77.1.7686915

[bib13] Kiyama S, Morrison K, Zellweger T, Akbari M, Cox M, Yu D, Miyake H, Gleave ME (2003) Castration-induced increases in insulin-like growth factor-binding protein 2 promotes proliferation of androgen-independent human prostate LNCaP tumors. Cancer Res 63: 3575–3584 12839944

[bib14] Kumar CC (1998) Signaling by integrin receptors. Oncogene 17: 1365–1373 9779984 10.1038/sj.onc.1202172

[bib15] Mehrian-Shai R, Chen CD, Shi T, Horvath S, Nelson SF, Reichardt JK, Sawyers CL (2007) Insulin growth factor-binding protein 2 is a candidate biomarker for PTEN status and PI3K/Akt pathway activation in glioblastoma and prostate cancer. Proc Natl Acad Sci USA 104: 5563–5568 17372210 10.1073/pnas.0609139104 PMC1838515

[bib16] Meinbach DS, Lokeshwar BL (2006) Insulin-like growth factors and their binding proteins in prostate cancer: cause or consequence? Urol Oncol 24: 294–306 16818181 10.1016/j.urolonc.2005.12.004

[bib17] Moore MG, Wetterau LA, Francis MJ, Peehl DM, Cohen P (2003) Novel stimulatory role for insulin-like growth factor binding protein-2 in prostate cancer cells. Int J Cancer 105: 14–19 12672024 10.1002/ijc.11015

[bib18] Murant SJ, Handley J, Stower M, Reid N, Cussenot O, Maitland NJ (1997) Co-ordinated changes in expression of cell adhesion molecules in prostate cancer. Eur J Cancer 33: 263–271 9135498 10.1016/s0959-8049(96)00418-2

[bib19] Perks CM, Holly JM (2008) IGF binding proteins (IGFBPs) and regulation of breast cancer biology. J Mammary Gland Biol Neoplasia 13: 455–469 19031049 10.1007/s10911-008-9106-4

[bib20] Perks CM, Vernon EG, Rosendahl AH, Tonge D, Holly JM (2007) IGF-II and IGFBP-2 differentially regulate PTEN in human breast cancer cells. Oncogene 26: 5966–5972 17369847 10.1038/sj.onc.1210397

[bib21] Pollak M (2008) Insulin, insulin-like growth factors and neoplasia. Best Pract Res Clin Endocrinol Metab 22: 625–638 18971123 10.1016/j.beem.2008.08.004

[bib22] Roddam AW, Allen NE, Appleby P, Key TJ, Ferrucci L, Carter HB, Metter EJ, Chen C, Weiss NS, Fitzpatrick A, Hsing AW, Lacey Jr JV, Helzlsouer K, Rinaldi S, Riboli E, Kaaks R, Janssen JA, Wildhagen MF, Schroder FH, Platz EA, Pollak M, Giovannucci E, Schaefer C, Quesenberry Jr CP, Vogelman JH, Severi G, English DR, Giles GG, Stattin P, Hallmans G, Johansson M, Chan JM, Gann P, Oliver SE, Holly JM, Donovan J, Meyer F, Bairati I, Galan P (2008) Insulin-like growth factors, their binding proteins, and prostate cancer risk: analysis of individual patient data from 12 prospective studies. Ann Intern Med 149: 461–471, W483-468 18838726 10.7326/0003-4819-149-7-200810070-00006 PMC2584869

[bib23] Schutt BS, Langkamp M, Rauschnabel U, Ranke MB, Elmlinger MW (2004) Integrin-mediated action of insulin-like growth factor binding protein-2 in tumor cells. J Mol Endocrinol 32: 859–868 15171717 10.1677/jme.0.0320859

[bib24] Schwartz MA (1997) Integrins, oncogenes, and anchorage independence. J Cell Biol 139: 575–578 9348275 10.1083/jcb.139.3.575 PMC2141711

[bib25] Skogseth H, Follestad T, Larsson E, Halgunset J (2006) Transcription levels of invasion-related genes in prostate cancer cells are modified by inhibitors of tyrosine kinase. APMIS 114: 364–371 16725013 10.1111/j.1600-0463.2006.apm_370.x

[bib26] Tamura M, Gu J, Danen EH, Takino T, Miyamoto S, Yamada KM (1999) PTEN interactions with focal adhesion kinase and suppression of the extracellular matrix-dependent phosphatidylinositol 3-kinase/Akt cell survival pathway. J Biol Chem 274: 20693–20703 10400703 10.1074/jbc.274.29.20693

[bib27] Thomas F, Holly JM, Persad R, Bahl A, Perks CM (2010) Fibronectin confers survival against chemotherapeutic agents but not against radiotherapy in DU145 prostate cancer cells: involvement of the insulin like growth factor-1 receptor. Prostate 70: 856–865 20127733 10.1002/pros.21119

[bib28] Thomas F, Patel S, Holly JM, Persad R, Bahl A, Perks CM (2009) Dihydrotestosterone sensitises LNCaP cells to death induced by epigallocatechin-3-Gallate (EGCG) or an IGF-I receptor inhibitor. Prostate 69: 219–224 18942120 10.1002/pros.20873

[bib29] Uzoh CC, Perks CM, Bahl A, Holly JM, Sugiono M, Persad RA (2009) PTEN-mediated pathways and their association with treatment-resistant prostate cancer. BJU Int 104: 556–561 19220271 10.1111/j.1464-410X.2009.08411.x

